# Liquor License

**Published:** 1876-02

**Authors:** 


### LIQUOR LICENSE.

ONE of the baldest hypocrisies of this age of sham and show, one of its completest stultifications, is exhibited in the legal permission of the sale of intoxicating drinks. Our city authorities, our judges, our lawyers, our legislators, are, in the main, a time-serving, fawning, unmanly race: knowing the right, they fear to enforce it, and prostitute their high intelligence to the meanest of all purposes, party aggrandizement.

Speaking of the degenerative influences now affecting mankind, *The British and Foreign Medico-Chirurgical Journal*, of January, says: "One of the most wide-spread of these degenerative influences is the habitual use of alcoholic fluids, constituting one of the most common causes of physical and mental disorder. Much of the anxiety and crime which men suffer is due to this lamentable propensity, and that which is detrimental to the individual, must become so to the race. That man must be blind, indeed, and morally perverted, who could pass a single day and night in a great metropolis, and not feel himself a wellsatisfied witness of this, humanity's great and most disgraceful shame. There is scarcely a more sad and depressing aspect under which a member of a Christian State can contemplate its government, than that which represents it as not tolerating, but absolutely fostering, houses for the sale of fermented fluids. Not sad and depressing alone, because of the evil and misery which result, but humiliating—because of the enormity of the hypocrisy, the intensity of the sham, which, on the one hand, providing laws and magistrates which meet and minister to the crime which drunkenness produces; on the other hand, draws its revenues from its very source, and does so much in a way to increase such revenue, that it becomes, not toleration only, but cordial support."

Such is the testimony which educated medicine bears uniformly against the habitual use of alcoholic drinks, and against their authorized sale. Those who advocate opposite views, do so from sheer ignorance of the question, or because they are already sufficiently predisposed to indulgences themselves, and they fear to inaugurate measures which may possibly cause them some self-denial.

